# EV-associated miRNAs from peritoneal lavage as potential diagnostic biomarkers in colorectal cancer

**DOI:** 10.1186/s12967-019-1954-8

**Published:** 2019-06-20

**Authors:** Berta Roman-Canal, Jordi Tarragona, Cristian Pablo Moiola, Sònia Gatius, Sarah Bonnin, Maria Ruiz-Miró, José Enrique Sierra, Maria Rufas, Esperanza González, José M. Porcel, Antonio Gil-Moreno, Juan M. Falcón-Pérez, Julia Ponomarenko, Xavier Matias-Guiu, Eva Colas

**Affiliations:** 1 Department of Pathology and Molecular Genetics/Oncologic Pathology Group, Biomedical Research Institute of Lleida (IRBLleida), University of Lleida, CIBERONC, Lleida, Spain; 2 Department of Pathology, University Hospital of Bellvitge, Bellvitge Biomedical Research Institute (IDIBELL), L’Hospitalet de Llobregat, Barcelona, Spain; 3 Universitat Autònoma de Barcelona (UAB), Barcelona, Spain; 4 Biomedical Research Group in Gynecology, Vall Hebron Research Institute (VHIR), Universitat Autònoma de Barcelona, CIBERONC, Pg. Vall Hebron 119-129, 08035 Barcelona, Spain; 5 Centre for Genomic Regulation (CRG), The Barcelona Institute or Science and Technology, Dr. Aiguader 88, Barcelona, 08003 Spain; 6 Department of Surgery, Hospital Arnau de Vilanova, Biomedical Research Institute of Lleida (IRBLleida), Lleida, Spain; 7 Exosomes Laboratory and Metabolomics Platform, CIC bioGUNE, CIBEREHD Bizkaia Technology Park, Derio, Spain; 8 Pleural Medicine Unit, Arnau de Vilanova University Hospital, IRBLleida, Lleida, Spain; 9 Gynecological Oncology Department, Vall Hebron University Hospital, CIBERONC, Barcelona, Spain; 10 IKERBASQUE, Basque Foundation for Science, 48011 Bilbao, Spain; 11 University Pompeu Fabra, Barcelona, Spain; 12 Oncologic Pathology Group, Department of Medicine UdL, Biomedical Research Institute of Lleida (IrbLleida), Av. Rovira Roure 80, 25198 Lleida, Spain

**Keywords:** Colorectal cancer, Biomarkers, Diagnostic, miRNAs, Ascitic fluid, Peritoneal lavage, Liquid biopsy, Extracellular vesicles, Colon cancer

## Abstract

**Background:**

Colorectal cancer (CRC) is the third leading cause of cancer-related mortality worldwide. Current systematic methods for diagnosing have inherent limitations so development of a minimally-invasive diagnosis, based on the identification of sensitive biomarkers in liquid biopsies could therefore facilitate screening among population at risk.

**Methods:**

In this study, we aim to develop a novel approach to identify highly sensitive and specific biomarkers by investigating the use of extracellular vesicles (EVs) isolated from the peritoneal lavage as a source of potential miRNA diagnostic biomarkers. We isolated EVs by ultracentrifugation from 25 ascitic fluids and 25 peritoneal lavages from non-cancer and CRC patients, respectively. Analysis of the expression of EV-associated miRNAs was performed using Taqman OpenArray technology through which we could detect 371 miRNAs.

**Results:**

210 miRNAs were significantly dysregulated (adjusted *p* value < 0.05 and abs(logFC) ≥ 1). The top-10 miRNAs, which had the AUC value higher than 0.95, were miRNA-199b-5p, miRNA-150-5p, miRNA-29c-5p, miRNA-218-5p, miRNA-99a-3p, miRNA-383-5p, miRNA-199a-3p, miRNA-193a-5p, miRNA-10b-5p and miRNA-181c-5p.

**Conclusions:**

This finding opens the avenue to the use of EV-associated miRNA of peritoneal lavages as an untapped source of biomarkers for CRC.

**Electronic supplementary material:**

The online version of this article (10.1186/s12967-019-1954-8) contains supplementary material, which is available to authorized users.

## Background

Colorectal cancer (CRC) is the third most common type of malignant tumor and the third leading cause of cancer-related mortality worldwide among men and women [1]. The overall survival of colorectal cancer is 65%, but this is highly dependent upon the disease stage at diagnosis, and ranges from a 90% of 5-year survival rate for cancers detected at the localized stage (40% of the cases) and 70% for regional (35% of the cases) to 15% for distant metastatic cancer (20% of the cases) [2]. Current systematic methods for diagnosis, such as fecal occult blood test and flexible sigmoidoscopy, help to reduce mortality by removing precursor lesions and making diagnosis at an earlier stage. However, these techniques have inherent limitations, such as low sensitivity and invasiveness for patients, respectively; and the burden of disease and mortality is still high [3]. Serum tumor markers CA19-9 and CEA have been used for detection of many types of cancer, but their sensitivity for the detection of CRC is low [4]. Therefore, development of a minimally-invasive diagnosis, based on the identification of sensitive biomarkers in liquid biopsies could therefore facilitate screening among population at risk of CRC, impact on early detection, and thus, decrease CRC-related mortality.

MiRNAs are a highly conserved family of endogenous non-coding and single-stranded RNAs that are 19–24 nucleotides in length [5]. Generally, miRNAs negatively regulate gene expression via binding to the 3′-untranslated region (3′-UTR) of their target double-stranded mRNA that results in transcriptional repression or mRNA degradation upon dicer complex [6]. miRNAs have been implicated in development and progression of CRC by functioning as oncogenes and tumor suppressors [7]. Recent studies demonstrated that miRNAs are secreted from various cells, including cancer cells, into bodily fluids such as blood, urine, breast milk, and saliva, either as free miRNAs or via extracellular vesicles (EVs) [4].

EVs are 20–200 nm membrane vesicles released by either directly from plasma membranes, or from intracellular multivesicular bodies by their fusion with the cell membrane. Their function is to mediate intercellular communication, influencing the recipient cell function. Importantly, EVs have awakened the interest of the scientific community as a source of biomarkers, mainly because they carry a broad range of bioactive material (proteins, metabolites, RNA, miRNA, etc.) and this material is well-protected owing to the EVs lipid bilayer membrane, even if EVs are extracted from circulating or proximal body fluids [8].

Herein, we investigated the use of EVs isolated from the peritoneal lavage, a proximal fluid in CRC patients, as a source of potential diagnostic biomarkers. To do so, we conducted miRNA-profiling of EVs isolated from peritoneal lavages of surgical CRC patients and ascitic fluids of non-cancer patients by using the TaqMan OpenArray Human MicroRNA Panel. We unveiled the most relevant individual miRNAs for diagnosing CRC and characterized the biological and molecular landscape of the CRC milieu. The study was conceived as a proof of concept investigation to demonstrate the feasibility of peritoneal lavage as a source of EV-associated miRNAs in patients with CRC.

## Methods

### Patients and ascitic fluid and peritoneal lavages collection

Participants in the study attended to the Hospital Arnau de Vilanova in Lleida, Spain. The Clinical Research Ethics Committee of the hospital approved the study and all the participating patients provided a signed informed consent. Ascitic fluids and peritoneal lavages were extracted from a cohort of 50 patients, corresponding to 25 control patients with decompensated cirrhosis, and 25 patients with CRC who underwent curative surgery. In control patients, the collection of ascitic fluid was aspirated using 18 or 21G needles (for diagnostic paracentesis) or an over-the-needle catheter device (for therapeutic paracentesis). The procedure was performed under sterile conditions, the site of needle insertion was selected by ultrasound guidance, and skin and parietal peritoneum were previously anesthetized with 2% mepivacine. A total of 100 mL of ascitic fluid was gently aspirated, collected into a 50 mL tube and stored at − 80 °C. In CRC patients, the collection of peritoneal lavage was performed before the surgery, once the abdominal cavity has been opened and prior to any manipulation of the colon. A total of 100 mL of physiological saline were instilled into the abdominal cavity with a 50 mL syringe, mobilizing patients for the correct distribution of saline, which was then extracted with a 50 mL syringe connected to a 14-gauge aspiration needle. The peritoneal lavage was gently aspirated. A volume ranging from 50 to 100 mL was collected and stored at − 80 °C. The clinical features of each patient are listed in Additional file 1: Table S1.

### EVs isolation

EVs were isolated with a differential centrifugation method as previously described [9] with slight modifications. Briefly, ascitic fluids and peritoneal lavages were centrifuged at 300×*g* for 10 min, followed by a centrifugation at 2500×*g* for 20 min and a centrifugation of 10,000*g* for 30 min (Thermo Scientific Heraeus MultifugeX3R Centrifuge (FiberLite rotor F15-8x-50c)). The supernatant was then filtered through 0.22 µm filters (Merck Millipore) and the sample obtained was transferred to ultracentrifuge tubes (Beckman Coulter) and filled with PBS to perform two consecutive ultracentrifugation steps at 100,000*g* for 2 h each on a Thermo Scientific Sorvall WX UltraSeries Centrifuge with an AH-629 rotor. The pellet containing the EVs was resuspended in 50 µL of PBS. From those, 5 µL were isolated for nanoparticle tracking analysis (NTA) and quantification, and the rest was frozen at − 80 °C with 500 µL of Qiazol for RNA extraction.

### Nanoparticle tracking analysis

Size and number of EVs was determined using a Nanosight LM10 instrument equipped with a 405 nm laser and a Hamamatsu C11440 ORCA-Flash 2.8 camera (Hamamatsu) with Nanoparticle Tracking Analysis (NTA, Malvern Instruments, UK). Each sample was diluted appropriately with Milli-Q water (Milli-Q Synthesis, Merck Millipore, Massachusetts, USA) to give counts in the linear range of the instrument. The particles in the laser beam undergo Brownian motion, and a video was recorded for 60 s in triplicate. Analysis was performed following manufacturer’s instructions and data were analyzed using the version 2.3 of the NTA-software.

### Total RNA extraction and OpenArray analysis

Total RNA, including miRNAs and other RNAs, was isolated from the EVs samples using the miRNeasy MiniKit (Qiagen) according to manufacturer’s protocol. RNA from EVs was eluted with 30 µL of Nuclease-free water (Ambion). MiRNA expression was performed using TaqMan OpenArray Human MicroRNA Panel, QuantStudio 12 K Flex (Catalog number: 4470187, Thermo Fisher Scientific), a fixed-content panel containing 754 well-characterized human miRNA sequences from the Sanger miRBase v14 and according to the manufacturer’s instructions. Reverse transcription (RT) was performed on 2 µL RNA using Megaplex™ Primer Pools A and B and the supporting TaqMan^®^ MicroRNA Reverse Transcription Kit as follows: 15 min at 42 °C and 5 min at 85 °C. Then, 5 µL of the resulting cDNA was preamplified prior to real-time PCR analysis using Megaplex™ PreAmp Pools and the TaqMan^®^ PreAmp Master Mix using the following conditions: one single step at 95 °C during 5 min, 20 cycles of a two-steps program (3 s, 95 °C and 30 s, 60 °C) followed by a single cycle of 10 min at 99 °C to inactivate the enzyme. The preamplified products were diluted 1:20 in 0.1× TE buffer pH8.0, and mixed in 1:1 with TaqMan^®^ OpenArray^®^ Real-Time PCR Master Mix in the 384-well OpenArray^®^ Sample Loading Plate. TaqMan^®^ OpenArray^®^ MicroRNA Panels were automatically loaded using the AccuFill™ System.

### Preprocessing and differential expression analysis

All bioinformatics analysis was performed with the BioConductor (version 3.7) [10] project in the R statistical environment (version 3.5.0) [11]. For the data preprocessing, the HTqPCR (version 1.34) R package [12] was used. Probes that had a “Cycle threshold” (Ct) value of 40 in all samples were removed. Further samples in which more than 80% of the probes had a Ct value above 40 were retained. To assure comparability across samples, the Ct values were delta normalized. The average Ct values of the probes *hsa *− *miR *− *150 *− *5p, hsa *− *let *− *7g*-*5p, hsa *− *miR *− *598 *− *3p*, and *hsa *− *miR *− *361 *− *3p* were used for normalization. These probes had the Ct values of 40 in a maximum of three samples, and the lowest interquartile range across samples. Differential expression analysis was carried out with an empirical Bayes approach on linear models, using the limma (version 3.36) R Package [13]. Results were corrected for multiple testing using the False Discovery Rate (FDR) [14].

### Development of predictors

For predictive analysis, the whole patient cohort was randomly divided into training and validation sets with the 3:2 ratio. Calculated (with the limma R Package) relative miRNA expression values were used as input variables to a logistic regression model between groups. Each miRNA (adjusted p-value < 0.05) was fitted in the logistic regression model to differentiate the CRC and the control patients groups in the training set and its classification ability was evaluated using the AUC (area under the ROC curve), accuracy, sensitivity, and specificity values on the validation set. The procedure from division into training and validation sets and fitting the logistic model was repeated 500 times and statistics were collected.

### miRNA target genes prediction and bioinformatics analysis

miRNAs target genes were obtained using the Predictive Target Module of miRWalk2.0 online software [15] (https://goo.gl/ajG9ja), considering the following parameters: 3´UTR localization, miRNA seed start at position 1 and minimum 7 bp seed length. To improve the accuracy of target gene prediction, only those transcripts that were predicted in at least 8 out of the 12 databases were considered (miRWalk, miRanda, MicroT4, miRDB, miRMap, miRBridge, miRNAMap, PICTAR2, RNA22, PITA, TargetScan, and RNAhybrid). Gene Ontology (GO) functional analysis were used to analyze the potential functions of the predicted target genes, using the online Panther software [16] (http://www.pantherdb.org/). Biological process (BP) and molecular function (MF) GO terms were analyzed and plotted.

## Results

We analyzed the miRNA profile of EVs isolated from the ascitic fluid of 25 control individuals and peritoneal lavage of 25 CRC patients. Additional file 2: Figure S1 illustrates the workflow that was followed in this study. The quality of EVs isolated from the ascitic fluids and peritoneal lavages was measured by size distribution and concentration by Nanoparticle Tracking Analysis, demonstrating that we analyzed a population mostly enriched in small EVs but also containing a low representation of larger vesicles (Additional file 3: Figure S2). MiRNAs were extracted from EVs for a systematic miRNA expression analysis using the Taqman OpenArray technology, through which we detected 371 out of the 754 miRNAs (49.2%) present in the OpenArray. Probes that had the Ct value of 40 in all samples and samples in which more than 80% of the probes had the Ct value above 40 were removed, resulting in 355 miRNAs from 22 control and 19 CRC patients analyzed for the differential expression analysis (Table 1).

**Table 1:** Clinicopathological characteristics of patients

	Colon Cancer	Control
Age		
Median	74	65
Minimum	50	52
Maximum	88	90
Gender		
Female	12	4
Male	7	18
Pathology		
Colon cancer	19	–
ADC low grade	15	–
ADC other types	4	–
Hepatic cirrhosis	–	20
Others	–	2

Clinical characteristics of the final cohort of patients included in the study after data normalization

*ADC* adenocarcinoma

**Fig. 1:**
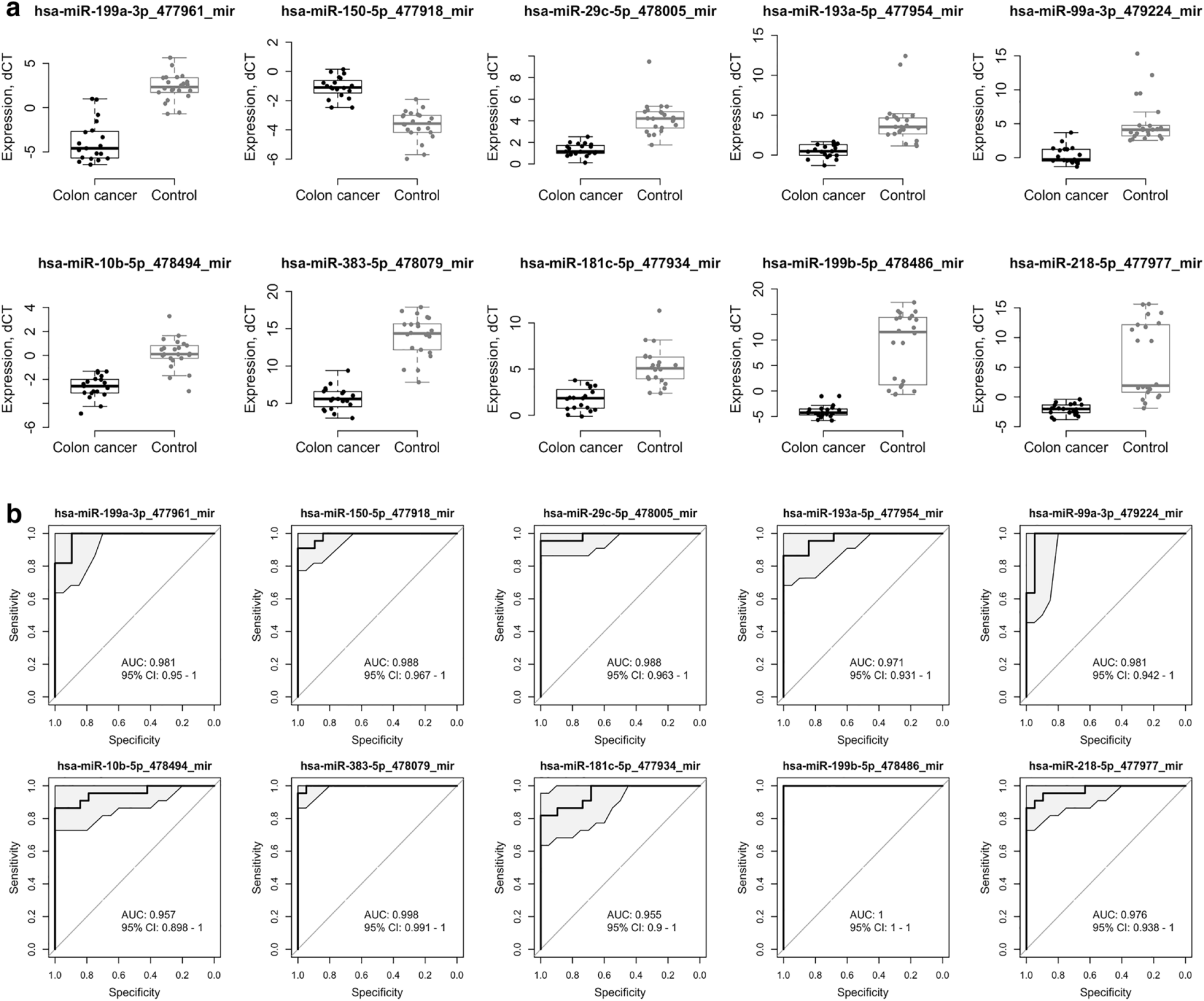
Diagnostic performance of the top-10 differentially expressed miRNAs. **a** Relative dCT values of top differentially expressed miRNAs in patients with CRC (n = 19) compared to control patients (n = 22). **p < 0.05. **b** ROC-curves and AUC-scores the top-10 differentially expressed miRNAs

The differential expression analysis between cancer and control cases yielded a list of 210 miRNAs that were significantly dysregulated (adj. p-value < 0.05 and logFC lower or higher than 1). Among those, 207 miRNA were found to be downregulated and 3 miRNA were upregulated in CRC patients. To evaluate whether these miRNAs can be used as diagnostics biomarkers, we performed a predictive analysis using the logistic modeling. Ten miRNAs demonstrated predictive performance at the AUC values higher than 0.95: miRNA-199b-5p, miRNA-150-5p, miRNA-29c-5p, miRNA-218-5p, miRNA-99a-3p, miRNA-383-5p, miRNA-199a-3p, miRNA-193a-5p, miRNA-10b-5p and miRNA-181c-5p (Table 2; Fig. 1). All those miRNAs were downregulated from 3.52 to 12.82 in the log_2_ scale with adjusted p-value lower than 1.56E−05, except miRNA-150-5p which was upregulated (adjusted p-value 3.41E−04). In Table 3, studies reporting an association between each of these top-10 miRNAs and CRC are described based on a search of Pubmed for each miRNA and the word “colorectal cancer”. Although there are some controversies among the different studies, most of the miRNA dysregulations observed in our study are concordant with the observations reported by other authors, either in tissue, plasma or stool samples. MiRNA-199b-5p, miRNA-29c-5p, and miRNA-99a-3p have never been reported previously in association to CRC.

**Table 2:** miRNA transcripts displaying a significant differential expression in patients with CRC compared to control patients

miRNA	LogFC	p-value	Adj. p-value	AUC	AUC 95% CI_lower	AUC 95% CI_upper	Accuracy	Sensitivity	Specificity
hsa-miR-199b-5p_478486_mir	− 12.82	2.59E−08	9.85E−07	1	1	1	0.967	0.968	0.964
hsa-miR-150-5p_477918_mir	2.58	7.10E−05	3.41E−04	0.978	0.959	0.996	0.919	0.936	0.899
hsa-miR-29c-5p_478005_mir	− 2.93	2.78E−08	9.85E−07	0.973	0.954	0.991	0.943	0.943	0.944
hsa-miR-218-5p_477977_mir	− 8.16	6.51E−07	8.25E−06	0.97	0.945	0.995	0.913	0.905	0.921
hsa-miR-99a-3p_479224_mir	− 4.89	1.51E−08	8.95E−07	0.97	0.95	0.99	0.94	0.976	0.9
hsa-miR-383-5p_478079_mir	− 8.33	3.55E−15	1.26E−12	0.968	0.952	0.985	0.939	0.94	0.938
hsa-miR-199a-3p_477961_mir	− 6.16	2.84E−09	2.65E−07	0.968	0.942	0.994	0.905	0.92	0.887
hsa-miR-193a-5p_477954_mir	− 3.62	1.32E−06	1.56E−05	0.962	0.932	0.991	0.873	0.852	0.897
hsa-miR-10b-5p_478494_mir	− 2.79	2.58E−07	4.17E−06	0.957	0.93	0.983	0.871	0.875	0.866
hsa-miR-181c-5p_477934_mir	− 3.52	1.23E−05	8.74E−05	0.952	0.929	0.974	0.833	0.859	0.803
hsa-miR-708-5p_478197_mir	− 6.45	9.72E−08	2.29E−06	0.946	0.917	0.975	0.877	0.834	0.926
hsa-miR-125b-5p_477885_mir	− 2.35	9.27E−07	1.13E−05	0.946	0.918	0.974	0.885	0.884	0.885
hsa-miR-140-5p_477909_mir	− 5.82	4.59E−05	2.39E−04	0.943	0.919	0.968	0.817	0.825	0.807
hsa-miR-451a_478107_mir	− 8.35	1.34E−07	2.64E−06	0.942	0.913	0.972	0.843	0.881	0.8
hsa-miR-148b-3p_477824_mir	− 3.05	1.42E−07	2.66E−06	0.942	0.916	0.968	0.834	0.853	0.813
hsa-miR-130a-3p_477851_mir	− 2.62	1.85E−06	2.00E−05	0.94	0.909	0.972	0.861	0.884	0.835
hsa-miR-214-3p_477974_mir	− 7.59	1.19E−07	2.49E−06	0.937	0.901	0.972	0.896	0.94	0.846
hsa-miR-10a-5p_479241_mir	− 2.19	1.44E−02	2.82E−02	0.937	0.906	0.969	0.897	0.904	0.889
hsa-miR-497-5p_478138_mir	− 3.68	2.13E−04	8.08E−04	0.936	0.911	0.961	0.814	0.829	0.797
hsa-miR-143-3p_477912_mir	− 3.15	1.58E−06	1.81E−05	0.936	0.906	0.965	0.86	0.868	0.85
hsa-miR-20a-5p_478586_mir	− 2.79	5.14E−08	1.30E−06	0.933	0.901	0.964	0.877	0.906	0.843
hsa-miR-29c-3p_479229_mir	− 3.55	2.14E−04	8.08E−04	0.931	0.897	0.965	0.86	0.84	0.883
hsa-miR-17-5p_478447_mir	− 3.68	4.83E−05	2.41E−04	0.93	0.893	0.966	0.874	0.898	0.847
hsa-miR-486-5p_478128_mir	− 11.10	2.21E−07	3.73E−06	0.929	0.899	0.958	0.853	0.818	0.893
hsa-miR-145-5p_477916_mir	− 3.07	6.47E−06	5.34E−05	0.929	0.899	0.958	0.877	0.901	0.851
hsa-miR-214-5p_478768_mir	− 8.13	2.33E−08	9.85E−07	0.923	0.885	0.96	0.877	0.906	0.844
hsa-miR-20b-5p_477804_mir	− 11.65	3.73E−09	2.65E−07	0.921	0.887	0.956	0.883	0.879	0.887
hsa-miR-551b-3p_478159_mir	− 9.71	2.07E−10	3.68E−08	0.919	0.885	0.953	0.852	0.906	0.79
hsa-miR-107_478254_mir	− 4.70	1.35E−03	3.73E−03	0.917	0.883	0.951	0.919	0.938	0.898
hsa-miR-202-5p_478755_mir	− 7.19	5.11E−08	1.30E−06	0.915	0.876	0.954	0.855	0.867	0.842
hsa-miR-93-5p_478210_mir	− 2.84	3.88E−04	1.30E−03	0.915	0.875	0.954	0.86	0.872	0.847
hsa-miR-483-3p_478122_mir	− 7.01	3.69E−09	2.65E−07	0.913	0.877	0.949	0.837	0.884	0.784
hsa-miR-652-3p_478189_mir	− 2.05	2.63E−05	1.70E−04	0.913	0.882	0.945	0.825	0.831	0.818
hsa-miR-29b-3p_478369_mir	− 3.59	3.71E−06	3.47E−05	0.911	0.877	0.945	0.836	0.834	0.838
hsa-miR-328-3p_478028_mir	− 5.03	2.86E−04	1.01E−03	0.908	0.874	0.941	0.819	0.798	0.842
hsa-miR-25-3p_477994_mir	− 2.37	3.44E−05	1.99E−04	0.908	0.87	0.946	0.865	0.899	0.827
hsa-miR-26a-5p_477995_mir	− 2.30	1.99E−05	1.38E−04	0.904	0.872	0.937	0.796	0.799	0.791
hsa-miR-296-5p_477836_mir	− 6.51	3.42E−05	1.99E−04	0.903	0.865	0.941	0.878	0.796	0.973
hsa-miR-144-3p_477913_mir	− 5.05	3.53E−05	1.99E−04	0.903	0.867	0.938	0.827	0.85	0.801
hsa-miR-769-5p_478203_mir	− 3.98	3.53E−05	1.99E−04	0.903	0.864	0.942	0.899	0.901	0.896
hsa-miR-181a-5p_477857_mir	− 2.13	5.21E−06	4.52E−05	0.902	0.865	0.939	0.843	0.895	0.783
hsa-miR-29a-3p_478587_mir	− 3.12	1.16E−03	3.38E−03	0.901	0.86	0.942	0.878	0.854	0.905
hsa-miR-152-3p_477921_mir	− 3.98	2.17E−05	1.48E−04	0.9	0.866	0.934	0.804	0.791	0.819
hsa-miR-125b-1-3p_478665_mir	− 9.13	1.93E−07	3.42E−06	0.895	0.86	0.931	0.86	0.868	0.851
hsa-miR-30a-3p_478273_mir	− 1.66	9.38E−05	4.27E−04	0.891	0.854	0.928	0.812	0.793	0.834
hsa-miR-449b-5p_479528_mir	− 10.10	2.30E−08	9.85E−07	0.889	0.846	0.932	0.88	0.908	0.847
hsa-miR-219a-5p_477980_mir	− 6.58	5.10E−07	7.54E−06	0.889	0.852	0.926	0.84	0.854	0.824
hsa-miR-125a-5p_477884_mir	− 1.40	1.48E−03	4.02E−03	0.888	0.846	0.93	0.827	0.84	0.813
hsa-miR-374b-3p_479421_mir	− 4.64	2.15E−06	2.24E−05	0.887	0.845	0.929	0.8	0.791	0.81
hsa-miR-101-3p_477863_mir	− 4.19	6.98E−05	3.39E−04	0.886	0.845	0.927	0.832	0.896	0.759
hsa-miR-452-5p_478109_mir	− 3.88	2.89E−05	1.80E−04	0.886	0.852	0.92	0.751	0.737	0.766
hsa-miR-193a-3p_478306_mir	− 2.46	2.37E−03	5.97E−03	0.884	0.841	0.928	0.869	0.899	0.835
hsa-miR-148a-3p_477814_mir	− 1.48	1.24E−03	3.52E−03	0.884	0.845	0.923	0.822	0.818	0.826
hsa-miR-133a-3p_478511_mir	− 6.89	1.67E−04	6.73E−04	0.883	0.843	0.922	0.827	0.853	0.797
hsa-miR-675-5p_478196_mir	− 4.22	5.46E−06	4.61E−05	0.883	0.845	0.92	0.766	0.721	0.817
hsa-miR-34a-5p_478048_mir	− 2.04	3.41E−05	1.99E−04	0.882	0.841	0.922	0.823	0.801	0.848
hsa-miR-582-5p_478166_mir	− 8.33	3.37E−07	5.20E−06	0.881	0.842	0.92	0.84	0.767	0.923
hsa-miR-2110_477971_mir	− 4.46	2.45E−06	2.48E−05	0.879	0.837	0.92	0.782	0.794	0.769
hsa-miR-185-5p_477939_mir	− 2.69	1.09E−02	2.18E−02	0.879	0.836	0.921	0.828	0.872	0.777
hsa-miR-144-5p_477914_mir	− 11.46	4.47E−08	1.30E−06	0.877	0.833	0.921	0.873	0.944	0.791
hsa-miR-199a-5p_478231_mir	− 7.83	1.24E−04	5.31E−04	0.877	0.831	0.923	0.772	0.712	0.84
hsa-miR-361-5p_478056_mir	− 1.48	9.56E−04	2.83E−03	0.877	0.837	0.918	0.821	0.86	0.778
hsa-miR-195-5p_477957_mir	− 2.80	2.19E−04	8.17E−04	0.875	0.832	0.919	0.835	0.822	0.849
hsa-miR-136-5p_478307_mir	− 8.01	5.88E−07	8.03E−06	0.873	0.834	0.913	0.828	0.821	0.836
hsa-miR-548d-5p_480870_mir	− 3.80	3.79E−03	8.96E−03	0.873	0.827	0.919	0.755	0.65	0.875
hsa-miR-30b-5p_478007_mir	− 2.55	2.64E−03	6.47E−03	0.873	0.829	0.916	0.774	0.795	0.751
hsa-miR-363-3p_478060_mir	− 8.98	3.58E−05	1.99E−04	0.869	0.825	0.914	0.782	0.776	0.789
hsa-miR-27b-3p_478270_mir	− 2.46	2.87E−02	4.90E−02	0.868	0.828	0.908	0.76	0.815	0.698
hsa-miR-24-3p_477992_mir	− 2.06	4.68E−05	2.40E−04	0.868	0.823	0.913	0.859	0.9	0.813
hsa-miR-499a-5p_478139_mir	− 4.05	4.42E−05	2.34E−04	0.864	0.821	0.908	0.851	0.875	0.824
hsa-miR-15a-5p_477858_mir	− 1.96	3.80E−04	1.28E−03	0.863	0.823	0.903	0.77	0.781	0.758
hsa-miR-31-3p_478012_mir	− 5.27	2.92E−04	1.02E−03	0.86	0.817	0.904	0.799	0.766	0.836
hsa-miR-18a-3p_477944_mir	− 4.70	5.55E−05	2.74E−04	0.859	0.817	0.901	0.761	0.747	0.777
hsa-miR-92a-3p_477827_mir	− 1.48	5.48E−04	1.74E−03	0.859	0.811	0.907	0.813	0.822	0.804
hsa-miR-130b-3p_477840_mir	− 5.57	3.72E−04	1.27E−03	0.858	0.815	0.901	0.748	0.644	0.866
hsa-let-7b-5p_478576_mir	− 1.57	5.76E−04	1.81E−03	0.858	0.814	0.902	0.76	0.758	0.761
hsa-miR-30e-3p_478388_mir	− 4.70	5.51E−03	1.22E−02	0.854	0.81	0.899	0.753	0.628	0.897
hsa-miR-23b-5p_477991_mir	− 3.62	3.36E−05	1.99E−04	0.853	0.81	0.896	0.756	0.758	0.754
hsa-miR-29b-2-5p_478003_mir	− 4.33	2.79E−06	2.75E−05	0.85	0.806	0.894	0.797	0.806	0.786
hsa-miR-30e-5p_479235_mir	− 8.36	6.16E−04	1.92E−03	0.849	0.802	0.896	0.709	0.638	0.791
hsa-miR-200c-3p_478351_mir	− 7.14	2.32E−05	1.53E−04	0.848	0.802	0.894	0.802	0.721	0.894
hsa-miR-1180-3p_477869_mir	− 3.91	4.43E−05	2.34E−04	0.847	0.798	0.896	0.806	0.754	0.866
hsa-miR-190a-5p_478358_mir	− 1.89	1.87E−02	3.48E−02	0.847	0.801	0.892	0.829	0.814	0.847
hsa-miR-151b_477811_mir	− 9.82	2.25E−04	8.31E−04	0.846	0.801	0.892	0.761	0.733	0.792
hsa-miR-505-5p_478957_mir	− 5.86	4.24E−05	2.32E−04	0.846	0.801	0.891	0.796	0.805	0.786
hsa-miR-196b-5p_478585_mir	− 6.46	1.86E−06	2.00E−05	0.845	0.795	0.894	0.767	0.67	0.879
hsa-miR-324-5p_478024_mir	− 1.51	8.13E−03	1.65E−02	0.843	0.797	0.889	0.777	0.754	0.802
hsa-miR-224-5p_477986_mir	− 2.72	1.58E−04	6.45E−04	0.842	0.8	0.883	0.746	0.695	0.804
hsa-miR-139-5p_478312_mir	− 5.12	2.43E−04	8.80E−04	0.839	0.794	0.885	0.727	0.725	0.729
hsa-miR-545-5p_479003_mir	− 5.30	8.42E−06	6.79E−05	0.838	0.79	0.886	0.79	0.79	0.79
hsa-miR-222-3p_477982_mir	− 2.08	7.82E−04	2.37E−03	0.838	0.791	0.884	0.728	0.762	0.689
hsa-miR-340-5p_478042_mir	− 9.05	9.31E−06	7.18E−05	0.836	0.79	0.882	0.803	0.817	0.786
hsa-miR-504-5p_478144_mir	− 3.54	1.86E−04	7.18E−04	0.836	0.787	0.886	0.778	0.729	0.833
hsa-miR-106a-5p_478225_mir	− 10.76	4.30E−08	1.30E−06	0.834	0.784	0.883	0.816	0.924	0.693
hsa-miR-1271-5p_478674_mir	− 9.10	3.87E−06	3.52E−05	0.834	0.785	0.883	0.823	0.9	0.734
hsa-miR-125b-2-3p_478666_mir	− 6.80	9.11E−06	7.18E−05	0.834	0.782	0.886	0.804	0.866	0.735
hsa-miR-339-3p_478325_mir	− 3.60	6.56E−03	1.39E−02	0.834	0.782	0.886	0.778	0.692	0.876
hsa-miR-483-5p_478432_mir	− 6.45	5.42E−07	7.69E−06	0.832	0.783	0.882	0.785	0.871	0.687
hsa-miR-584-5p_478167_mir	− 10.36	6.40E−07	8.25E−06	0.831	0.781	0.882	0.803	0.906	0.685
hsa-miR-17-3p_477932_mir	− 8.43	2.22E−05	1.49E−04	0.831	0.779	0.883	0.805	0.861	0.741
hsa-miR-570-3p_479053_mir	− 4.46	1.11E−04	4.79E−04	0.831	0.785	0.876	0.766	0.697	0.844
hsa-miR-625-5p_479469_mir	− 10.93	4.36E−06	3.87E−05	0.83	0.781	0.88	0.806	0.865	0.738
hsa-miR-196a-5p_478230_mir	− 7.37	1.01E−05	7.64E−05	0.83	0.777	0.883	0.812	0.879	0.735
hsa-miR-7-1-3p_478198_mir	− 7.37	1.00E−04	4.52E−04	0.829	0.783	0.874	0.762	0.646	0.895
hsa-miR-450b-5p_478914_mir	− 9.65	1.03E−07	2.29E−06	0.828	0.775	0.88	0.803	0.906	0.685
hsa-miR-221-5p_478778_mir	− 5.01	3.08E−04	1.06E−03	0.827	0.779	0.875	0.745	0.64	0.865
hsa-miR-128-3p_477892_mir	− 1.32	5.21E−03	1.16E−02	0.823	0.772	0.874	0.743	0.741	0.746
hsa-miR-491-5p_478132_mir	− 3.50	1.88E−03	4.92E−03	0.822	0.774	0.87	0.743	0.703	0.79
hsa-miR-136-3p_477902_mir	− 7.94	2.78E−05	1.76E−04	0.821	0.771	0.871	0.786	0.855	0.707
hsa-miR-101-5p_478620_mir	− 7.44	1.09E−05	8.08E−05	0.819	0.766	0.873	0.812	0.877	0.738
hsa-miR-151a-3p_477919_mir	− 1.93	2.85E−04	1.01E−03	0.819	0.769	0.87	0.803	0.854	0.745
hsa-miR-28-3p_477999_mir	− 2.25	4.30E−03	1.00E−02	0.817	0.771	0.863	0.738	0.728	0.749
hsa-miR-489-3p_478130_mir	− 4.40	1.33E−04	5.62E−04	0.815	0.766	0.865	0.76	0.736	0.787
hsa-miR-106b-3p_477866_mir	− 2.41	6.48E−03	1.39E−02	0.815	0.765	0.866	0.72	0.693	0.751
hsa-miR-324-3p_478023_mir	− 7.87	2.85E−04	1.01E−03	0.814	0.766	0.861	0.761	0.686	0.847
hsa-miR-125a-3p_477883_mir	− 3.24	4.18E−04	1.39E−03	0.811	0.76	0.862	0.765	0.741	0.791
hsa-let-7i-3p_477862_mir	− 6.66	4.69E−04	1.54E−03	0.81	0.759	0.861	0.747	0.665	0.841
hsa-miR-33b-5p_478479_mir	− 5.49	2.06E−03	5.30E−03	0.81	0.758	0.862	0.704	0.626	0.793
hsa-miR-503-5p_478143_mir	− 2.80	6.04E−03	1.31E−02	0.81	0.757	0.863	0.762	0.726	0.805
hsa-miR-301a-3p_477815_mir	− 5.08	1.51E−03	4.05E−03	0.809	0.76	0.858	0.723	0.608	0.856
hsa-miR-330-3p_478030_mir	− 5.72	7.79E−04	2.37E−03	0.805	0.754	0.856	0.761	0.645	0.892
hsa-miR-425-5p_478094_mir	− 1.51	2.66E−02	4.63E−02	0.805	0.757	0.852	0.728	0.714	0.744
hsa-miR-16-2-3p_477931_mir	− 3.32	1.12E−02	2.22E−02	0.804	0.747	0.862	0.78	0.769	0.793
hsa-miR-548k_479374_mir	− 14.17	8.73E−03	1.76E−02	0.801	0.757	0.845	0.718	0.654	0.79
hsa-miR-429_477849_mir	− 2.00	1.76E−02	3.37E−02	0.801	0.747	0.854	0.768	0.807	0.723
hsa-miR-598-3p_478172_mir	− 1.51	4.51E−03	1.04E−02	0.8	0.754	0.847	0.696	0.726	0.661
hsa-miR-887-3p_479189_mir	− 5.41	1.42E−04	5.95E−04	0.799	0.748	0.85	0.737	0.632	0.858
hsa-miR-93-3p_478209_mir	− 4.60	2.28E−04	8.33E−04	0.798	0.745	0.852	0.749	0.702	0.801
hsa-miR-629-5p_478183_mir	− 6.16	1.75E−04	6.90E−04	0.796	0.746	0.846	0.753	0.678	0.839
hsa-miR-21-5p_477975_mir	1.32	7.91E−03	1.61E−02	0.796	0.751	0.842	0.683	0.688	0.677
hsa-miR-140-3p_477908_mir	− 3.42	6.10E−03	1.31E−02	0.793	0.743	0.843	0.734	0.752	0.713
hsa-miR-425-3p_478093_mir	− 5.28	9.90E−04	2.90E−03	0.792	0.74	0.844	0.728	0.588	0.888
hsa-miR-200a-5p_478752_mir	3.05	2.34E−02	4.15E−02	0.792	0.735	0.85	0.713	0.693	0.736
hsa-miR-590-3p_478168_mir	− 4.77	1.27E−03	3.56E−03	0.791	0.739	0.842	0.702	0.637	0.776
hsa-miR-30a-5p_479448_mir	− 8.52	7.71E−04	2.37E−03	0.789	0.738	0.841	0.734	0.726	0.744
hsa-let-7 g-3p_477850_mir	− 6.38	1.74E−04	6.90E−04	0.787	0.733	0.84	0.756	0.608	0.926
hsa-miR-542-3p_478153_mir	− 8.15	2.88E−06	2.76E−05	0.786	0.729	0.842	0.778	0.901	0.638
hsa-miR-31-5p_478015_mir	− 1.78	7.34E−03	1.51E−02	0.786	0.737	0.835	0.676	0.708	0.64
hsa-miR-379-5p_478077_mir	− 5.15	5.46E−04	1.74E−03	0.78	0.724	0.835	0.755	0.773	0.734
hsa-miR-194-5p_477956_mir	− 1.66	1.21E−03	3.48E−03	0.78	0.724	0.836	0.778	0.861	0.683
hsa-miR-34c-5p_478052_mir	− 2.51	4.47E−03	1.04E−02	0.779	0.726	0.832	0.685	0.639	0.738
hsa-miR-576-5p_478165_mir	− 6.85	8.70E−05	4.01E−04	0.778	0.721	0.836	0.774	0.853	0.684
hsa-miR-28-5p_478000_mir	− 5.87	1.45E−03	3.96E−03	0.778	0.726	0.829	0.731	0.64	0.835
hsa-miR-708-3p_479162_mir	− 2.77	2.27E−03	5.75E−03	0.772	0.715	0.828	0.738	0.74	0.736
hsa-miR-505-3p_478145_mir	− 2.12	3.62E−03	8.67E−03	0.771	0.718	0.824	0.758	0.737	0.781
hsa-miR-26b-5p_478418_mir	− 1.01	2.37E−02	4.19E−02	0.768	0.717	0.819	0.692	0.714	0.666
hsa-miR-365a-3p_478065_mir	− 4.75	2.67E−02	4.63E−02	0.767	0.713	0.822	0.661	0.495	0.85
hsa-miR-423-3p_478327_mir	− 1.63	1.58E−03	4.19E−03	0.765	0.716	0.815	0.65	0.687	0.607
hsa-miR-338-5p_478038_mir	− 3.61	2.40E−03	6.01E−03	0.761	0.703	0.819	0.704	0.719	0.687
hsa-miR-210-3p_477970_mir	− 1.17	1.67E−02	3.22E−02	0.761	0.708	0.815	0.672	0.698	0.643
hsa-miR-551a_478158_mir	− 5.58	1.97E−03	5.10E−03	0.76	0.699	0.822	0.741	0.69	0.8
hsa-miR-889-3p_478208_mir	− 8.68	1.14E−05	8.28E−05	0.759	0.698	0.82	0.789	0.922	0.637
hsa-miR-301b-3p_477825_mir	− 5.75	1.87E−02	3.48E−02	0.758	0.7	0.816	0.751	0.851	0.637
hsa-miR-590-5p_478367_mir	− 3.83	1.63E−02	3.15E−02	0.757	0.703	0.812	0.707	0.59	0.841
hsa-miR-548am-5p_480872_mir	− 3.25	1.13E−02	2.22E−02	0.757	0.703	0.81	0.663	0.526	0.82
hsa-miR-187-3p_477941_mir	− 4.47	6.97E−03	1.46E−02	0.754	0.701	0.807	0.704	0.644	0.772
hsa-miR-450a-5p_478106_mir	− 7.12	1.26E−03	3.56E−03	0.753	0.692	0.814	0.743	0.646	0.853
hsa-miR-376a-5p_478859_mir	− 5.94	1.04E−04	4.56E−04	0.75	0.688	0.811	0.738	0.832	0.631
hsa-miR-1296-5p_479451_mir	− 4.26	2.49E−03	6.17E−03	0.75	0.695	0.806	0.703	0.719	0.686
hsa-miR-181c-3p_477933_mir	− 3.64	9.29E−04	2.77E−03	0.748	0.685	0.811	0.715	0.805	0.613
hsa-miR-1247-5p_477882_mir	− 3.36	2.43E−02	4.26E−02	0.748	0.692	0.804	0.698	0.586	0.827
hsa-miR-34a-3p_478047_mir	− 2.32	1.77E−02	3.37E−02	0.748	0.694	0.803	0.658	0.727	0.579
hsa-miR-654-3p_479135_mir	− 7.56	4.77E−04	1.55E−03	0.74	0.681	0.799	0.76	0.692	0.839
hsa-miR-411-5p_478086_mir	− 5.12	7.16E−03	1.49E−02	0.738	0.68	0.796	0.715	0.647	0.793
hsa-miR-181d-5p_479517_mir	− 5.67	4.10E−03	9.63E−03	0.733	0.675	0.791	0.686	0.554	0.838
hsa-miR-200b-3p_477963_mir	− 3.43	7.26E−03	1.50E−02	0.733	0.674	0.792	0.683	0.609	0.767
hsa-miR-299-3p_478792_mir	− 6.51	4.73E−05	2.40E−04	0.732	0.67	0.794	0.748	0.893	0.582
hsa-miR-182-5p_477935_mir	− 8.68	8.37E−05	3.91E−04	0.73	0.666	0.795	0.753	0.9	0.585
hsa-miR-410-3p_478085_mir	− 5.65	1.22E−03	3.48E−03	0.728	0.67	0.787	0.753	0.771	0.733
hsa-miR-744-5p_478200_mir	− 3.55	1.81E−02	3.40E−02	0.725	0.67	0.781	0.657	0.637	0.68
hsa-miR-96-5p_478215_mir	− 6.84	1.03E−04	4.56E−04	0.724	0.658	0.79	0.744	0.886	0.583
hsa-miR-133b_480871_mir	− 3.18	1.98E−02	3.66E−02	0.724	0.664	0.783	0.686	0.651	0.726
hsa-miR-544a_478156_mir	− 5.67	1.37E−03	3.77E−03	0.719	0.66	0.779	0.756	0.81	0.694
hsa-miR-497-3p_478946_mir	− 6.92	1.44E−04	5.95E−04	0.718	0.652	0.784	0.753	0.899	0.585
hsa-miR-331-3p_478323_mir	− 5.93	3.27E−03	7.90E−03	0.715	0.656	0.774	0.761	0.775	0.744
hsa-let-7f-2-3p_477843_mir	− 3.26	1.19E−02	2.33E−02	0.715	0.655	0.776	0.682	0.567	0.814
hsa-miR-195-3p_478744_mir	− 4.54	6.67E−03	1.40E−02	0.712	0.652	0.773	0.717	0.712	0.723
hsa-miR-378a-5p_478076_mir	− 5.30	2.12E−03	5.41E−03	0.71	0.65	0.769	0.723	0.584	0.882
hsa-miR-1-3p_477820_mir	− 6.27	6.10E−03	1.31E−02	0.706	0.644	0.768	0.732	0.778	0.679
hsa-miR-615-3p_478175_mir	− 3.14	4.99E−03	1.13E−02	0.706	0.643	0.769	0.696	0.652	0.746
hsa-miR-545-3p_479002_mir	− 2.99	1.99E−02	3.66E−02	0.704	0.64	0.767	0.675	0.678	0.672
hsa-miR-548a-3p_478157_mir	− 3.75	4.83E−03	1.10E−02	0.703	0.641	0.765	0.716	0.572	0.881
hsa-miR-1248_478653_mir	− 2.64	1.08E−02	2.17E−02	0.702	0.642	0.763	0.707	0.764	0.643
hsa-miR-381-3p_477816_mir	− 5.10	5.97E−03	1.31E−02	0.701	0.639	0.763	0.734	0.727	0.742
hsa-miR-627-5p_478427_mir	− 4.94	5.01E−04	1.62E−03	0.701	0.632	0.77	0.735	0.863	0.589
hsa-miR-1301-3p_477897_mir	− 5.30	5.52E−03	1.22E−02	0.696	0.638	0.754	0.711	0.596	0.843
hsa-miR-486-3p_478422_mir	− 6.30	1.86E−04	7.18E−04	0.692	0.626	0.758	0.725	0.891	0.535
hsa-miR-200a-3p_478490_mir	− 4.37	1.79E−02	3.39E−02	0.691	0.626	0.756	0.719	0.792	0.635
hsa-miR-15a-3p_477928_mir	− 3.53	6.65E−03	1.40E−02	0.688	0.62	0.755	0.703	0.808	0.584
hsa-miR-548 g-3p_479020_mir	− 2.12	2.25E−02	4.04E−02	0.687	0.625	0.75	0.691	0.759	0.613
hsa-miR-432-5p_478101_mir	− 3.66	2.70E−02	4.65E−02	0.683	0.621	0.746	0.691	0.724	0.652
hsa-miR-15b-5p_478313_mir	− 6.53	2.89E−02	4.90E−02	0.682	0.619	0.745	0.666	0.692	0.637
hsa-miR-132-5p_478705_mir	− 6.01	3.23E−03	7.86E−03	0.68	0.614	0.745	0.734	0.867	0.582
hsa-let-7e-5p_478579_mir	− 5.40	2.04E−02	3.72E−02	0.679	0.614	0.744	0.655	0.556	0.769
hsa-miR-485-5p_478126_mir	− 2.09	2.46E−02	4.30E−02	0.678	0.613	0.743	0.688	0.734	0.634
hsa-miR-299-5p_478793_mir	− 5.05	8.70E−04	2.62E−03	0.672	0.603	0.741	0.702	0.858	0.524
hsa-miR-16-1-3p_478727_mir	− 4.44	1.55E−03	4.13E−03	0.669	0.603	0.735	0.699	0.841	0.537
hsa-miR-215-5p_478516_mir	− 7.23	8.19E−05	3.88E−04	0.668	0.601	0.735	0.718	0.896	0.514
hsa-miR-103a-2-5p_477864_mir	− 4.32	2.05E−02	3.73E−02	0.664	0.603	0.724	0.664	0.529	0.818
hsa-miR-29a-5p_478002_mir	− 6.48	2.54E−03	6.26E−03	0.643	0.568	0.717	0.723	0.896	0.524
hsa-miR-874-3p_478205_mir	− 2.80	2.77E−02	4.74E−02	0.638	0.575	0.701	0.617	0.524	0.722
hsa-miR-502-5p_478954_mir	− 4.03	2.18E−02	3.93E−02	0.637	0.573	0.701	0.662	0.53	0.813
hsa-miR-542-5p_478337_mir	− 5.28	3.71E−03	8.83E−03	0.633	0.564	0.702	0.68	0.855	0.481
hsa-miR-362-3p_478058_mir	− 5.81	1.73E−03	4.56E−03	0.632	0.564	0.701	0.69	0.879	0.475
hsa-miR-431-3p_478888_mir	− 4.55	4.63E−03	1.06E−02	0.624	0.552	0.696	0.678	0.853	0.478
hsa-miR-192-3p_478741_mir	− 4.08	5.15E−03	1.16E−02	0.61	0.535	0.685	0.674	0.85	0.474
hsa-miR-589-5p_479073_mir	− 2.81	2.28E−02	4.07E−02	0.582	0.512	0.652	0.633	0.795	0.448
hsa-miR-888-5p_479192_mir	− 3.79	2.06E−02	3.73E−02	0.559	0.489	0.63	0.656	0.861	0.422
hsa-miR-15b-3p_477929_mir	− 3.73	1.76E−02	3.37E−02	0.529	0.459	0.599	0.629	0.86	0.365
hsa-miR-651-5p_479131_mir	− 3.94	2.90E−02	4.90E−02	0.523	0.448	0.599	0.638	0.87	0.372

Log fold-change expression, p-value, adjusted p-value, AUC values, accuracy, sensitivity, specificity, and 95% of confidence intervals of the 210 dysregulated miRNAs

*CI* confidence of interval

**Table 3:** Published studies of the top-10 miRNAs dysregulated in CRC patients

	Tissue samples	Other type of samples
miR-199b-5p	Not previously reported	Not previously reported
miR-150-5p	Upregulated: [24, 25]	Downregulated: serum [28]
	Downregulated: [26, 27]	
miR-29c-5p	Not previously reported	Not previously reported
miR-218-5p	Upregulated: [29]	Not previously reported
	Downregulated: [30, 31]	
miR-99a-3p	Not previously reported	Not previously reported
miR-383-5p	Downregulated: [32]	Not previously reported
miR-199a-3p	Upregulated: [26]	Upregulated in stool [33, 34]
miR-193a-5p	Downregulated in CRC cell lines [35]	Not previously reported
miR-10b-5p	Downregulated: [30, 36, 37]	Not previously reported
miR-181c-5p	Upregulated: [38]	Not previously reported

**Fig. 2:**
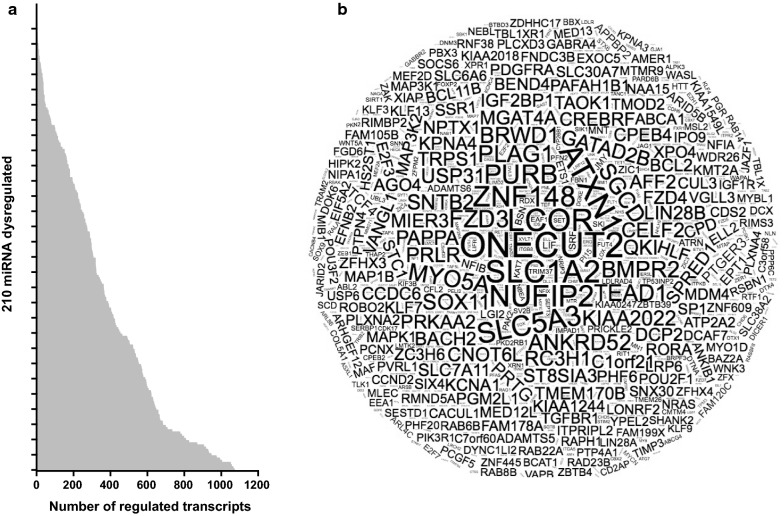
Prediction of miRNA target transcripts. **a** Boxplot of the number of transcripts regulated by the 210 dysregulated miRNAs. **b** Graphical representation in which the transcript ID is represented with a different font size accordingly to the times that is predicted to be regulated by the different miRNAs, i.e. the most frequently regulated transcripts are shown in a larger font size

**Fig. 3:**
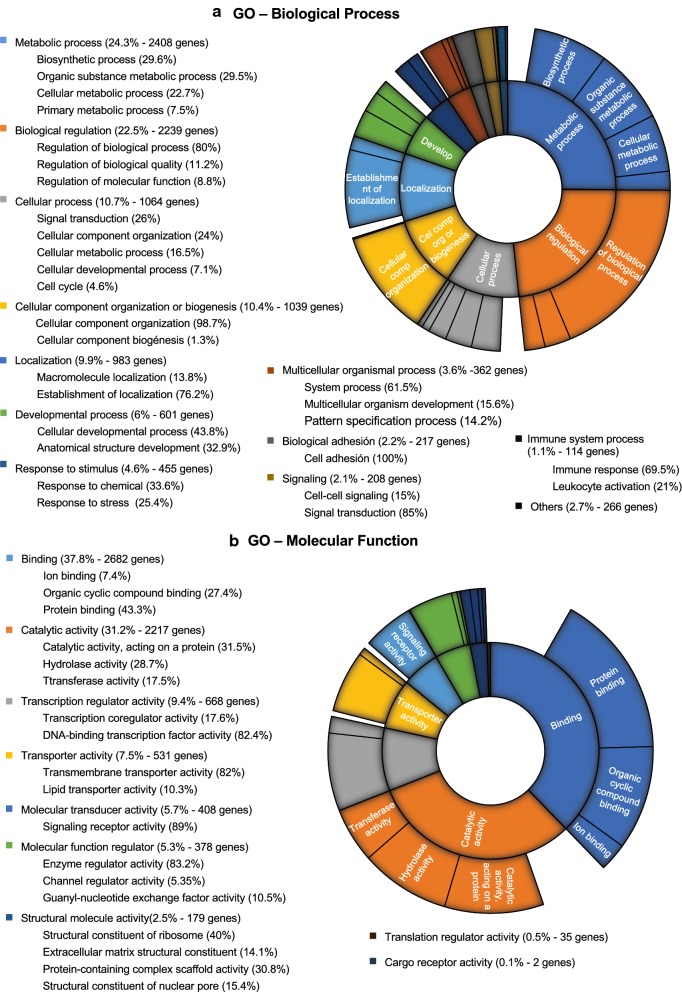
Sun projection plot of GO terms. Predicted transcripts regulated by the differentially expressed miRNAs. **a** GO analysis of up-regulated and down-regulated target genes according to biological process. **b** GO analysis of up-regulated and down-regulated target genes according to molecular function

To further understand the milieu generated by CRC EVs, we performed a bioinformatics study to first unveil the predicted transcripts that are regulated by all the differential miRNAs, and then assess the biological processes and molecular functions in which they participate. A total of 9358 transcripts were found to be regulated by the 210 miRNA differentially expressed. Figure 2 shows the number and most frequently regulated transcripts predicted for each dysregulated miRNA. To comprehensively integrate the properties of all target transcripts, they were studied using Gene Ontology (GO). The most enriched biological processes in CRC EVs were metabolic processes (24.3%), mostly including biosynthetic process, organic substance metabolomic process and cellular metabolic process; biological regulation (22.5%); cellular processes (10.7%), signal transduction, cellular component organization and cellular metabolic process; and cellular component organization or biogenesis, including cellular component organization (Fig. 3a). In relation to the most altered molecular functions in CRC EVs, the Gene Ontology (GO) analysis revealed that many targeted transcripts were found to be involved in binding (37.8%), including protein binding and organic cyclic compound binding; and in catalytic activity (31.2%), including catalytic activity, and protein and hydrolase activity (Fig. 3b).

## Discussion

In this study we investigated, for the first time, the miRNA content of EVs isolated from peritoneal lavages and ascitic liquid of CRC and control patients, respectively. Our study shows that EV-associated miRNAs can be consistently extracted from peritoneal lavages and ascitic liquids and that miRNA expression profiles can indicate and represent the status of CRC patients. The EV-associated miRNA were analyzed by Taqman OpenArray technology and the differential expression analysis yielded a list of 210 miRNAs that were significantly dysregulated in CRC patients, being downregulated the 98.57% of the altered miRNAs.

The finding that miRNAs are dysregulated in CRC patients is known, as many studies have reported this previously, mostly in tissue specimens [17] but also in different body fluids. In CRC, most of the studies use plasma or serum as it is the most common, easy-to-handle, accessible liquid biopsy. The first report detected 69 miRNAs in serum of CRC patients but not in serum of normal controls [18]. Since then, several studies have identified miRNA upregulation or downregulation in plasma or serum samples [17] including studies that have focus on the search of biomarkers in miRNAs dysregulated in the vesicular fraction of the serum or plasma of CRC patients. Hiroko Ogata-Kawata et al. [4] analyzed the EV-associated miRNA profile of serum samples from CRC patients and healthy controls and identified 16 miRNA that were expressed in a significantly higher levels among CRC patients. Of these, 7 miRNAs (let-7a, miR-1229, miR-1246, miR-150, miR-21, miR-223, and miR-23a) were suggested as promising diagnostic biomarkers of CRC with an AUC between 0.67 and 0.95. More recently, the serum exosomal miRNA-19a was found to be upregulated in the serum of CRC patients compared to healthy volunteers, but also was associated with poor prognosis [19]. Finally, Zhao et al. [20], demonstrated that the exosomal miRNA-21 expression is associated with the early diagnosis of CRC. Although plasma and serum have reported promising biomarkers for CRC diagnosis, other approaches as it is the use of proximal bodily fluids as a source of biomarkers have aroused the attention of the biomarker research community. Proximal bodily fluids, such as urine for prostate cancer [21], or uterine fluid for endometrial cancer [22] have demonstrated that this type of fluids offers an improved representation of the molecular alterations that takes place in the tumor. The peritoneal lavage is a proximal fluid with an unexploded value in biomarker research for cancers originating within the peritoneal cavity. Tokuhisa et al. [23] showed that EV-associated miRNAs can be consistently extracted from this bodily fluid and that miRNAs expression profiles can indicate the status of peritoneum in gastric cancer patients.

To the best of our knowledge, our study is the first to report the value of this proximal fluid for the identification of miRNAs associated to EVs in CRC. Importantly, this study unveiled the promising use of the top-10 miRNA dysregulated (miRNA-199b-5p, miRNA-150-5p, miRNA-29c-5p, miRNA-218-5p, miRNA-99a-3p, miRNA-383-5p, miRNA-199a-3p, miRNA-193a-5p, miRNA-10b-5p and miRNA-181c-5p) as diagnostic biomarkers, all showing the AUC value higher than 0.95. Those biomarkers should be validated as well as combined in order to increase the already excellent accuracy of individual miRNAs. However, this should be done in an independent study including a larger cohort of patients. Moreover, further analysis should be performed to elucidate the prognostic value of the detection of the different types of miRNAs in EVs isolated from peritoneal lavages.

## Conclusions

In this study, we have demonstrated that use of EV-associated miRNA of ascitic liquid from control patients and peritoneal lavages from CRC patients are an untapped source of biomarkers. Specifically, we identified miRNA-199b-5p, miRNA-150-5p, miRNA-29c-5p, miRNA-218-5p, miRNA-99a-3p, miRNA-383-5p, miRNA-199a-3p, miRNA-193a-5p, miRNA-10b-5p and miRNA-181c-5p as promising biomarkers of CRC diagnosis with the AUC value higher than 0.95.

## Additional files

**Additional file 1: Table S1.** Clinicopathological characteristics of all patients.

**Additional file 2: Figure S1.** Workflow. Workflow of the study design.

**Additional file 3: Figure S2.** EVs characterization. (**A**) Box-plot representing the average mode of EVs isolated from the peritoneal lavage and ascitic fluid of CRC and control patients, respectively (Mean ± SD); measured by Nanoparticle Tracking Analysis. (**B**) Size distribution and concentration of isolated EVs of a peritoneal lavage of a CRC patient (left) and a ascitic fluid of a control patient (right), measured by Nanoparticle Tracking Analysis.

## Data Availability

All bioinformatics analysis was performed with the BioConductor (version 3.7) [10] project in the R statistical environment (version 3.5.0).

## Abbreviations

CRC: colorectal cancer
EVs: extracellular vesicles
RT: reverse transcription
Ct: cycle threshold
FDR: False Discovery Rate
AUC: area under the ROC curve
GO: Gene Ontology
BP: biological process
MF: molecular function
3′-UTR: 3′-untranslated region
