# Supplementary figures and images for: EV-associated miRNAs from peritoneal lavage as potential diagnostic biomarkers in colorectal cancer
Source: J Transl Med. 2019 Jun 20;17:208. doi: 10.1186/s12967-019-1954-8 (PMC6585099; doi:10.1186/s12967-019-1954-8)

Additional file 2: Figure S1

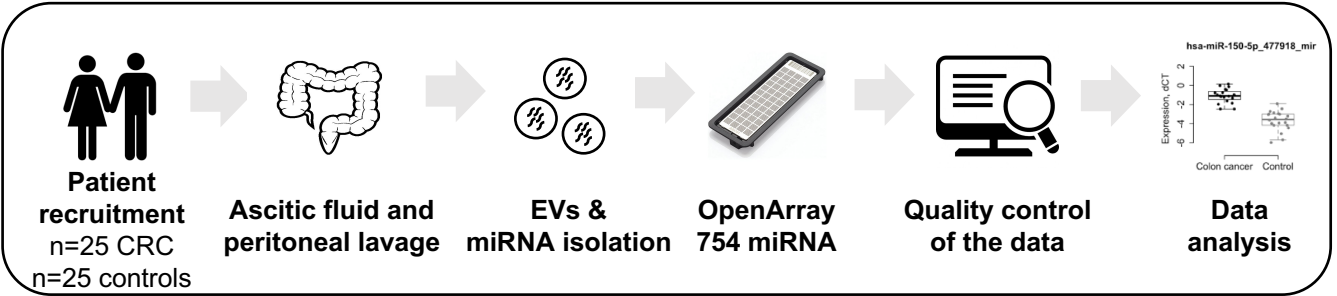

Supplement: Supplementary file 2 — Additional file 2: Figure S1. Workflow. Workflow of the study design. [file 12967_2019_1954_MOESM2_ESM.pdf]

Additional file 3: Figure S2

A

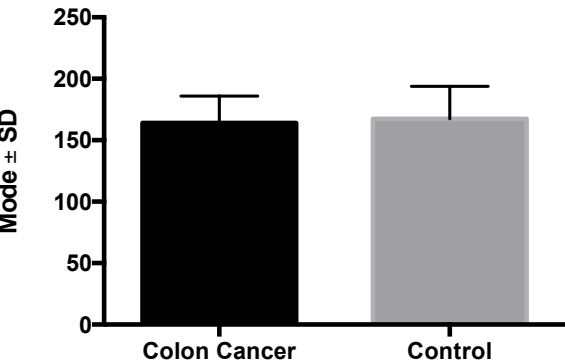

B

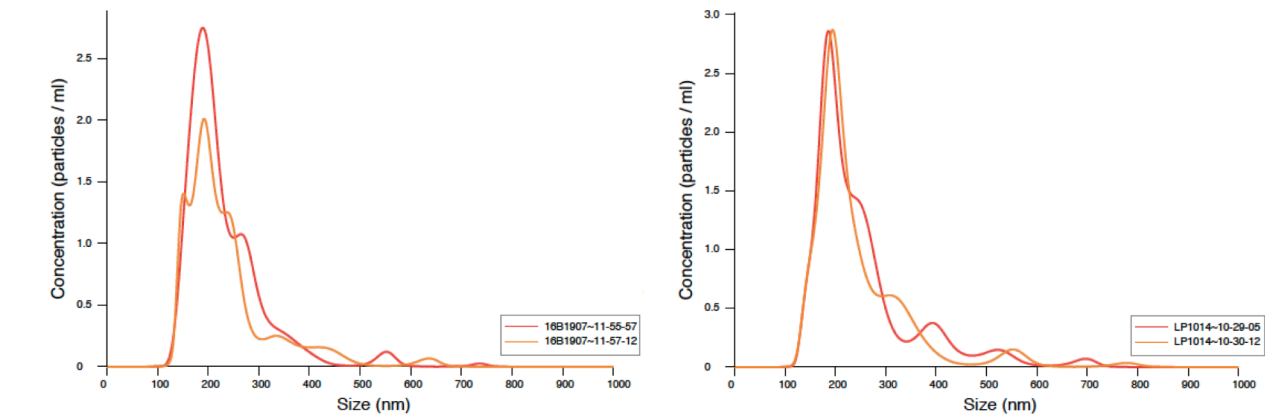

Supplement: Supplementary file 3 — Additional file 3: Figure S2. EVs characterization. (A) Box-plot representing the average mode of EVs isolated from the peritoneal lavage and ascitic fluid of CRC and control patients, respectively (Mean ± SD); measured by Nanoparticle Tracking Analysis. (B) Size distribution and concentration of isolated EVs of a peritoneal lavage of a CRC patient (left) and a ascitic fluid of a control patient (right), measured by Nanoparticle Tracking Analysis. [file 12967_2019_1954_MOESM3_ESM.pdf]
